# Association between Acrylamide Metabolites and Cardiovascular Risk in Children With Early Stages of Chronic Kidney Disease

**DOI:** 10.3390/ijms21165855

**Published:** 2020-08-14

**Authors:** Chien-Ning Hsu, Chih-Yao Hou, Pei-Chen Lu, Guo-Ping Chang-Chien, Sufan Lin, You-Lin Tain

**Affiliations:** 1 Department of Pharmacy, Kaohsiung Chang Gung Memorial Hospital, Kaohsiung 833, Taiwan; cnhsu@cgmh.org.tw; 2 School of Pharmacy, Kaohsiung Medical University, Kaohsiung 807, Taiwan; 3 Department of Seafood Science, National Kaohsiung University of Science and Technology, Kaohsiung 811, Taiwan; chihyaohou@webmail.nkmu.edu.tw; 4 Department of Pediatrics, Kaohsiung Chang Gung Memorial Hospital and College of Medicine, Chang Gung University, Kaohsiung 833, Taiwan; alexiellu@gmail.com; 5 Center for Environmental Toxin and Emerging-Contaminant Research, Cheng Shiu University, Kaohsiung 833, Taiwan; guoping@csu.edu.tw (G.-P.C.-C.); linsufan2003@csu.edu.tw (S.L.); 6 Super Micro Mass Research and Technology Center, Cheng Shiu University, Kaohsiung 833, Taiwan; 7 Institute for Translational Research in Biomedicine, Kaohsiung Chang Gung Memorial Hospital and College of Medicine, Chang Gung University, Kaohsiung 833, Taiwan

**Keywords:** acrylamide, ambulatory blood pressure monitoring, cardiovascular disease, children, chronic kidney disease, citrulline, hypertension, nitric oxide, symmetric dimethylarginine

## Abstract

Cardiovascular disease (CVD) begins early in children with chronic kidney disease (CKD). Reduced nitric oxide (NO) bioavailability has been associated with increased CVD in CKD patients. Children tend to have more exposure to acrylamide, one of the most common toxins in food. We aimed to determine whether urinary levels of acrylamide metabolites N-acetyl-S-(2-carbamoylethyl)-cysteine (AAMA) and N-acetyl-S-(2-carbamoyl-2-hydroxyethyl)-cysteine (GAMA) are associated with CV risk markers in children with CKD. Data on 112 children and adolescents ages three to 18 years old with CKD stage G1–G4 are reported. We observed that 24 h ambulatory blood pressure monitoring (ABPM) abnormalities were greater, and left ventricular (LV) mass and ambulatory arterial stiffness index (AASI) were higher in children with CKD stage G2–G4 versus G1. Patients with CKD stage G2–G4 had a lower urinary acrylamide level, but a higher AAMA-to-GAMA ratio than those with CKD stage G1. Urinary acrylamide level was negatively associated with high systolic blood pressure (SBP) and diastolic BP (DBP) load on 24 h ABPM. Lower urinary levels of acrylamide, AAMA, and GAMA were correlated with LV mass. Additionally, GAMA are superior to AAMA related to NO-related parameters, namely citrulline and symmetric dimethylarginine (SDMA). This study suggests that determinations of urinary acrylamide level and its metabolites in the early stages of pediatric CKD may identify patients at risk of CVD. Further studies should clarify mechanisms underlying acrylamide exposure to define the treatment for protection against CVD.

## 1. Introduction

Chronic kidney disease (CKD) is highly prevalent for both adults and children [1,2]. Cardiovascular disease (CVD) is the leading cause of morbidity and mortality among patients with CKD [3,4]. Unlike adults, overt features of CVD are rarely present in children. Hypertension is the most important risk factor for premature CVD [5]. More than 50% of children with early stages of CKD exhibited blood pressure (BP) abnormalities on 24 h ambulatory BP monitoring (ABPM) [6,7]. Additionally, several surrogate markers have been applied to stratify the cardiovascular risk in CKD children, including left ventricular (LV) mass, LV mass index (LVMI), and ambulatory arterial stiffness index (AASI) [8].

Acrylamide, an industrially produced α, β-unsaturated carbonyl-containing volatile organic compound, is one of the most common toxins in food [9,10]. It occurs in food containing high concentrations of hydrocarbons subjected to high temperature, like breads, potato chips, and coffee [10]. Once absorbed, acrylamide can be converted to glycidamide or conjugated with glutathione (GSH). Acrylamide and glycidamide have a short half-life. The majority of absorbed acrylamide is eliminated via the urine after conversion to mercapturic acid derivatives. GSH conjugates of acrylamide and glycidamide are further converted to mercapturic acid derivatives, N-acetyl-S-(2-carbamoylethyl)-cysteine (AAMA), and N-acetyl-S-(2-carbamoyl-2-hydroxyethyl)-cysteine (GAMA), respectively [11]. Accordingly, urinary AAMA and GAMA levels have become widely accepted internal biomarkers of acrylamide exposure [11]. Acrylamide has raised much concern for its negative health effects, such as carcinogenicity, neurotoxicity, reproductive toxicity, and induction of oxidative stress [10]. However, study of the effect of acrylamide exposure on hypertension and CVD is limited, especially in pediatric population. Our previous report demonstrated that in CKD children, abnormal BP was associated with an impaired nitric oxide (NO) pathway [6]. The interplay between NO and oxidative stress has been linked to hypertension and CKD [12,13], whereas whether acrylamide exposure affects the NO pathway and relates to the CV risk in CKD children is largely unknown. We, therefore, aimed to estimate the associations of urinary acrylamide metabolites with BP and CV risk markers in children with CKD. We also investigated the mediating roles of the NO pathway in the relationships between urinary acrylamide metabolites and CV outcomes to identify the potential mechanistic link.

## 2. Results

### 2.1. Patient Characteristics

The characteristics of the 112 study participants are reported in Table 1. This cohort was a median age of 11.4 years, 58.9% male, and 65.2% with congenital anomalies of the kidney and urinary tract (CAKUT). Structural anomalies within the CAKUT spectrum include renal agenesis, kidney hypo/dysplasia, multicystic kidney dysplasia, horseshoe kidney, obstructive uropathy, reflux nephropathy, polycystic kidney disease, and ureter abnormalities. We included children and adolescents with CKD stage G1–G4, including 77 G1 subjects (75.5%), 27 G2 subjects (25.2%), seven G3 subjects (6.9%), and one G4 subject (1%). CKD children and adolescents were stratified according to the estimated glomerular filtration rate (eGFR) and divided into two groups: the G1 group (eGFR ≥ 90 mL/min/1.73 m^2^) and the G2–G4 group (eGFR < 90 mL/min/1.73 m^2^). CKD G2–G4 patients were older, predominantly male, and had higher systolic BP, blood urea nitrogen, creatinine (Cr), and uric acid, but lower eGFR than those with stage G1. Approximately 48% (49/102) of CKD children were diagnosed as having hypertension based on clinical BP measurement. However, there was no difference between the two groups in this characteristic.

### 2.2. Urinary Acrylamide Metabolites

**Figure 1:**
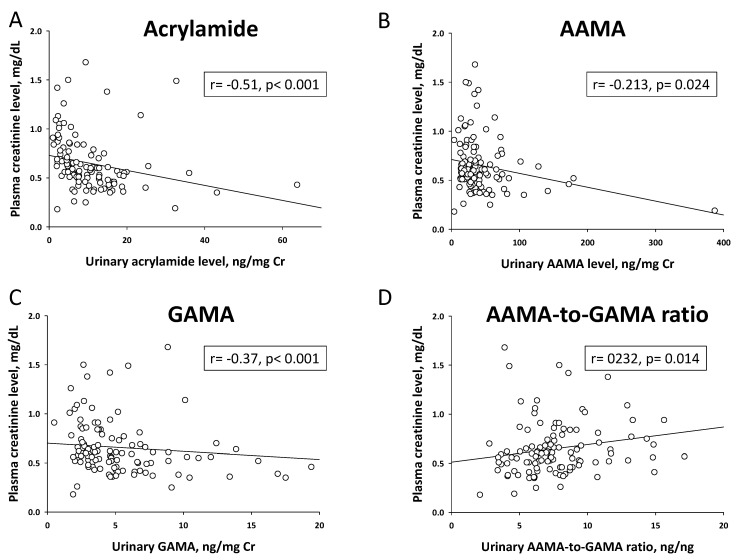
Correlation of urinary levels of (**A**) acrylamide (ng/mg Cr), (**B**) AAMA (ng/mg Cr), (**C**) GAMA (ng/mg Cr), and (**D**) AAMA-to-GAMA ratio (ng/ng) with plasma creatinine level by Spearman’s correlation coefficient. AAMA = N-acetyl-S-(2-carbamoylethyl)-cysteine. GAMA = N-acetyl-S-(2-carbamoyl-2-hydroxyethyl)-cysteine.

As presented in Table 2, urinary acrylamide level was lower in children with CKD G2–G4 than those with G1. Urinary AAMA and GAMA are generally accepted internal biomarkers of acrylamide exposure [11]. Our data showed there was no difference of urinary AAMA and GAMA levels between the two groups. The ratio of AAMA and GAMA has been considered as a metric to describe the extent of conversion of acrylamide to glycidamide [11]. In the present study, children with CKD G2–G4 had a higher AAMA-to-GAMA ratio than those with CKD stage G1. Analyses that evaluated the existence of associations between urinary levels of acrylamide metabolites and renal function, as assessed by data pooled from all subjects, indicated that there were significantly inverse correlations between plasma Cr level and urinary acrylamide (Figure 1A, *r =* −0.51, *p* < 0.001), AAMA (Figure 1B, *r =* −0.213, *p* = 0.024), and GAMA (Figure 1C, *r =* −0.37, *p* < 0.001), while plasma Cr level was positively correlated with urinary AAMA-to-GAMA ratio (Figure 1D, *r =* 0.232, *p* = 0.014).

### 2.3. Plasema NO-Related Parameters

Table 3 displays the plasma levels of NO-related parameters in children and adolescents with CKD. We found significant differences between the two groups on plasma citrulline (the precursor of arginine) and symmetric dimethylarginine (SDMA, an endogenous inhibitor of NO synthase) levels, with the CKD G2–G4 group showing higher levels. There was no difference of plasma arginine (the substrate for NO synthase) and asymmetric dimethylarginine (ADMA, an endogenous inhibitor of NO synthase) levels, and arginine-to-ADMA ratio between the two groups.

### 2.4. Cardiovascular Assessment

For these analyses, 74 participants aged over six years wore an ABPM for 24 h and received echocardiographic examination. In children and adolescents with CKD, 53% (39/74) of them had at least one BP load abnormality on ABPM, including seven subjects (9.5%) with 24 h BP or daytime BP > 95th percentile, eight subjects (10.8%) with nighttime BP > 95th percentile, 22 subjects (29.7%) with BP load ≥ 95th percentile, and 30 patients (40.5%) with a non-dipping nocturnal BP profile (Table 4). The cases with 24 h, daytime, and nighttime BP > 95th percentile and BP load ≥ 25% were greater in CKD stage G2–G4 compared with those in the G1 group. Using the readings of ABPM, the AASI was defined as 1 minus the regression slope of the diastolic BP (DBP) on systolic BP (SBP) [14], to evaluate arterial stiffness [15]. Moreover, the LV mass and LVMI were calculated using images obtained from echocardiographic examination. We observed that the AASI and LV mass were higher in children with CKD stage G2–G4 versus G1, while LVMI was not different between the two groups. Moreover, Spearman’s rank correlation analysis showed a strong positive association between LV mass with 24 h SBP load (*r =* 0.532, *p* < 0.001) and 24 h DBP load (*r =* 0.407, *p* < 0.001) on ABPM. Likewise, LVMI was correlated with 24 h SBP load (*r =* 0.279, *p* = 0.016) and 24 h DBP load (*r =* 0.234, *p* = 0.045).

### 2.5. Association between Acrylamide Metabolites and Cardiovascular Risk Markers

Across all CKD patients, correlations between urinary acrylamide metabolite levels and CV risk markers were analyzed (Table 5). Spearman’s rank correlation analysis revealed the urinary acrylamide level was negatively correlated with 24 h SBP (*r =* −0.368, *p* = 0.001), daytime SBP (*r =* −0.373, *p* = 0.001), nighttime SBP (*r =* −0.356, *p* = 0.002), 24 h DBP (*r =* −0.32, *p* = 0.005), daytime DBP (*r =* −0.31, *p* = 0.007), nighttime DBP (*r =* −0.234, *p* = 0.045), and LV mass (*r =* −0.566, *p* < 0.001). Additionally, urinary AAMA (*r =* −0.34, *p* < 0.001) and GAMA levels (*r =* −0.559, *p* < 0.001) displayed negative correlations, but AAMA-to-GAMA ratio (*r =* 0.261, *p* = 0.005) exhibited a positive correlation with LV mass. AASI was not correlated to almost all acrylamide metabolites, except with the AAMA-to-GAMA ratio (*r =* 0.252, *p* = 0.03).

Analysis of urinary acrylamide levels stratified according to the ABPM profile showed the acrylamide level was significantly lower in CKD children with high BP load, non-night dipping, and abnormal ABPM profile (Table 6). Analysis of AAMA, GAMA, and AAMA-to-GAMA ratio did not reveal any significant difference between CKD children with abnormal and normal ABPM profile.

Then we performed multivariate linear regression analyses to specify the exact role of each urinary acrylamide metabolite biomarker in the NO pathway and CV risk (Table 7). A multivariate linear regression model using stepwise selection was applied for age, sex, eGFR, and other acrylamide metabolites. In the best predictive model (*r =* 0.45, *p* = 0.001), plasma citrulline level was associated with urinary AAMA (*p* = 0.006), GAMA (*p* < 0.001), and the AAMA-to-GAMA ratio (*p* = 0.022). Urinary GAMA level was associated with plasma SDMA level controlling for age (*r =* 0.39, *p* = 0.015). These findings suggest a strong influence of acrylamide exposure on the NO pathway. Additionally, an association between AAMA (*p* = 0.015), GAMA (*p* = 0.014), and the AAMA-to-GAMA ratio (*p* = 0.041) and LVMI was found in the adjusted regression model controlling for eGFR and age (*r =* 0.472, *p* < 0.001).

## 3. Discussion

To our knowledge, this is the first study describing the association of urinary acrylamide metabolites with cardiovascular risk markers in a pediatric CKD population. Our study shows (1) children with CKD stage G2–G4 had a lower urinary acrylamide level, but a higher AAMA-to-GAMA ratio than those with CKD stage G1; (2) urinary acrylamide level was negatively associated with high SBP and DBP load on ABPM and plasma Cr level; (3) LV mass had a negative correlation with urinary acrylamide, AAMA, and GAMA levels, but a positive correlation with the AAMA-to-GAMA ratio; and (4) GAMA is superior to AAMA related to NO-related parameters, mainly citrulline and SDMA, in CKD children.

In keeping with previous studies, certain markers of CV risk tend to present in children with CKD, even in an early stage, such as ABPM abnormalities [4,6,7,16], uric acid [17], and AASI [18]. In the current study, up to 47.9% of children with CKD stage G1 displayed abnormal ABPM profiles. Our data support the notion that children in early stages of CKD are frequently masked by office BP measurements, and ABPM is recommended for the diagnosis of hypertension, especially in children at high cardiovascular risk [7,8,19].

Children were reported to have a higher acrylamide intake than adults [9]. In line with a previous study showing median urinary levels of AAMA and GAMA were 36.0 and 13.4 μg/L in children [20], our results showed AAMA was 26.8 and GAMA was 4.1 μg/L (before normalization by Cr) in children and adolescents with CKD. Interestingly, urinary levels of acrylamide, AAMA, and GAMA were negatively correlated with plasma Cr level in the current study. It is presumably due to lower excretion of acrylamide metabolites in children with more advanced CKD. Additionally, we found only urinary acrylamide, but not AAMA and GAMA, levels were inversely associated with SBP and DBP load on ABPM. These findings suggest CKD children have a lower acrylamide conversion capacity or a lower excretion rate, leading to a higher internal exposure which may increase the risk of developing high BP.

Consistent with previous studies of pediatric CKD [7,21,22], our data support the finding that left ventricular hypertrophy (LVH) is common and is associated with ABPM abnormalities in children with CKD, even in an early stage. In the current study, urinary acrylamide, AAMA and GAMA levels were negatively correlated with LV mass. Acrylamide exposure was reported to induce cardiotoxicity in animal studies [23,24], thus low urinary levels of acrylamide metabolites related to LVH in CKD children is likely to reflect decreased acrylamide excretion, or conversion may be a risk factor for adverse CV outcome. AASI is an index of arterial stiffness, which related to abnormal ABPM profile and LV mass in CKD children, tying well with our previous studies [6,22]. As expected, the severity of CKD is associated with high AASI and LV mass in the current study.

Notably, the positive associations between CV risk markers, LV mass, AASI, and the AAMA-to-GAMA ratio were observed in our study. The urinary AAMA-to-GAMA ratio is a measure of the extent of conversion of acrylamide to glycidamide and a high ratio reflects more acrylamide is conjugated with GSH than converted to glycidamide [25]. It seems likely that acrylamide rather than glycidamide may be the substantial contributor to CV risk in CKD children. Since the toxicity of acrylamide is related to reducing GSH and provoking oxidative stress [26], and GSH is beneficial in reducing CV risk in CKD [27], whether this ratio may reflect the redox status and correlate with other oxidative stress markers in CKD children awaits further clarification.

In line with our previous studies [6,28], which showed that NO-related parameters correlated with ABPM abnormalities and CV risk markers, this present study further extends their associations with acrylamide metabolites in CKD children. Our data demonstrated that plasma citrulline level was associated with urinary levels of AAMA and GAMA, and the AAMA-to-GAMA ratio. Besides, urinary GAMA level was correlated with plasma SDMA level. In CKD, renal citrulline uptake is diminished, the amount of citrulline converted to arginine in the kidney is reduced, and plasma citrulline levels and turnover are elevated [29]. SDMA is an endogenous NOS inhibitor and its level is elevated in advanced CKD [30]. Accordingly, high citrulline and SDMA levels represent reduced NO bioavailability in favor of elevating BPs. Our data support the impaired NO pathway as possibly the major factor contributing to hypertension and CV risk in children with CKD. Noteworthy, the association of citrulline and SDMA with GAMA were found to be stronger than with AAMA, suggesting a probably stronger oxidative stress potency of glycidamide toward the impaired NO pathway than acrylamide. Because this study is the first to report the association between acrylamide metabolites and NO parameters, more studies are warranted to elucidate the mechanisms underlying acrylamide-induced impairment of NO-dependent CV risk in patients with CKD.

Our study has some limitations. First, we did not recruit non-CKD controls because we examined the difference of CV risk markers between two levels of renal function (i.e., CKD stage G1 vs. stage G2–G4). That is, children with CKD stage G1 were served as the controls in the present study. Although urinary levels of acrylamide metabolites determined in the present study are comparable to those previously published in children [20], whether exposure levels of acrylamide are different between children with and without CKD remains to be determined. Second, we used ABPM reference from a different ethnic group [31]. Although several indices of CV risk markers we used in the current study have been examined in children, their age-specific reference ranges are still lacking [8]. Thus, our results may not be applicable to other ethnic populations until further reproducibility studies are conducted. Last, statistical comparisons were performed in a small cohort from one hospital and would not be representative of an entire population. Future multicenter studies with large sample sizes may be required to detect the true relationship.

## 4. Materials and Methods

### 4.1. Patients and Study Design

This was an observational cohort study in pediatric patients with CKD approved by the Institution Review Board and Ethics Committee of Chang Gung Medical Foundation, Taoyuan, Taiwan (Permit number: 201701735A3C5011; approval date: 3 October 2018) and was therefore performed in accordance with the 1964 Helsinki Declaration and its later amendments. Inclusion criteria were ages 3–18 years old and CKD stage 1–4. The CKD stage was defined by the Kidney Disease Improving Global Outcomes (KDIGO) 2012 clinical practice guideline [32]. The estimated glomerular filtration rate (eGFR) was calculated with the Schwartz formula, based on body height and blood Cr level [33]. CKD children were categorized according to eGFR (mL/min/1.73 m^2^): G1 ≥ 90, G2 60–89, G3 30–59, or G4 15–29. The exclusion criteria were existing congenital heart disease, pregnancy, eGFR < 15 mL/min/1.73 m^2^, dialysis, renal transplantation, and not cooperating with CV assessment. Written informed consent from participants was obtained prior to study enrollment. This ancillary study was performed in a subgroup of 112 participants who received the analysis of acrylamide metabolites. Fasting blood samples were drawn and aliquoted, and spot urine samples were collected and stored at −80 °C in a freezer until analysis. Blood urea nitrogen, Cr, uric acid, glucose, hemoglobin, total cholesterol, low-density lipoprotein (LDL), triglyceride, sodium, potassium, calcium, phosphate, and urine total protein-to-creatinine ratio were measured by the hospital central laboratory as described previously [6]. Plasma citrulline, arginine, ADMA and SDMA levels were analyzed with our validated method using a high-performance liquid chromatography (HPLC; HP series 1100; Agilent Technologies Inc., Santa Clara, CA, USA) with the O-phthalaldehyde 3-mercaptopropionic acid (OPA-3MPA) derivatization reagent [6]. Homoarginine (Sigma-Aldrich, St. Louis, MO, USA) was used as the internal standard.

### 4.2. High-Performance Liquid Chromatography–Mass Spectrometry Analysis

Determination of urinary acrylamide, AAMA, and GAMA was performed by a high-performance liquid chromatography system (Agilent 1260 Infinity II; Agilent Technologies, Santa Clara, CA) coupled with electrospray tandem mass spectrometry (Agilent Triple Quad 6470; Agilent Technologies, Santa Clara, CA, USA) following a previously published method with minor modifications [34]. In this procedure, 65 μL of urine sample was mixed with 35 μL of 103 ng/mL ^13^C_3_-acrylamide, AAMA-D_3_, and GAMA-D_3_ as internal standards and 900 μL ultrapure water, vortexed and centrifuged at 14,000 rpm for 5 min. Supernatant was then injected for analysis. The MS/MS was operated in positive multiple reaction mode (MRM) by monitoring the following ion mass transitions for quantitation of acrylamide and GAMA: acrylamide, m/z 72→55; ^13^C_3_-acrylamide, m/z 75→58; GAMA, m/z 251→146; and GAMA-D_3_, m/z 254→146. The negative MRM was operated for quantitation of AAMA by monitoring ion mass transitions m/z 233→104 for AAMA and m/z 236→104 for AAMA-D_3_. All correlation coefficients of these calibration curves for acrylamide, GAMA, and AAMA exceeded 0.995. Calibration curves were constructed with pooled urine samples spiked to the concentration range of 1–200 ng/mL for acrylamide, GAMA, and AAMA. The limits of detection (LODs) and quantitation (LOQs) were 0.4 and 1.0 ng/mL for acrylamide, AAMA and GAMA, respectively. The relative recoveries of target compounds ranged from 85 to 105%, with a coefficient of variation of 10%. The urinary concentration of each metabolite was corrected for urine Cr concentration, which was represented in ng/mg Cr.

### 4.3. Blood Pressure Measurement and Echocardiography

Participants were instructed to measure office BP at the clinic visit after an initial 5 min of rest. Three seated, non-dominant arm BP measurements were recorded at 1 min intervals. The ABPM was performed over a 24 h period using an Oscar II monitoring device (SunTech Medical, Morrisville, NC, USA) for subjects aged 6–18 years. The BP cuff was worn with the cuff size determined by upper arm circumference, and an in-office test measurement was performed by an experienced specialist nurse to assess adequate fit and comfort handled [6]. The device was programmed to take measurements at 20 min intervals from 7 am to 10 pm and at 30 min intervals from 10 pm to 7 am. The participants and their parents were requested to complete a diary to define sleep and awake periods and to note activities that may influence BP measurements. An abnormal ABPM profile was defined as (1) daytime, nighttime, systolic, or diastolic BPs ≥ 95th percentile according to gender and height; (2) daytime, nighttime, systolic or diastolic BP load ≥ 25%; and (3) nighttime BP load dipping < 10% using ABPM reference data [31]. The AASI was defined as 1 minus the slope of the diastolic on systolic BP during ABPM [14]. Echocardiographic examination was performed by pediatric cardiologists using a Philips IE33 system machine (Philips, Bothell, WA, USA). The LV mass was calculated using images obtained in the parasternal long-axis or short-axis view of the left ventricle by M-mode echocardiography. The LVMI was obtained by indexing LV mass to height^2.7^ [35].

### 4.4. Statistical Analysis

Results are presented with the median (25th–75th percentile) or number (%). All statistical analyses were conducted using the Statistical Package for the Social Sciences (SPSS) software 14.0 (Chicago, IL, USA). The Mann–Whitney *U*-test or Chi-square test was used to test the differences in variables between the two groups. The associations between variables were examined using Spearman’s rank correlation coefficient. A linear regression model was performed, followed by the stepwise multivariable analyses integrating relevant parameters to explain NO-related parameters and LVMI. Differences were considered significant at a probability level of *p* < 0.05.

## 5. Conclusions

In conclusion, our findings demonstrate that decreased urinary levels of acrylamide metabolites AAMA and GAMA but an increased AAMA-to-GAMA ratio are associated with cardiovascular risk markers in a pediatric CKD population. Our results cast new light on the link between the acrylamide metabolites, NO pathway, and cardiovascular risk in children with early stages of CKD. Given that children tend to be more exposed to acrylamide from daily-consumed food, further research is warranted to corroborate our findings and reveal the underlying mechanisms behind the adverse effects of acrylamide to improve cardiovascular outcomes in childhood CKD.

## Figures and Tables

**Table 1:** Clinical anthropometric and biomedical characteristics of 112 children and adolescents with chronic kidney disease (CKD).

CKD Stage	G1	G2–G4
Characteristics	*n* = 77	*n* = 35
Age, years	10.8 (7.2–14)	13.7 (6.7–17.5) *
Male	38 (49.4%)	28 (80%) *
Body height, percentile	50 (20–75)	25 (25–75)
Body weight, percentile	50 (15–85)	25 (15–85)
Body mass index, kg·m^−2^	17.9 (15.1–20.9)	18.6 (15.6–23.1)
Systolic blood pressure, mmHg	109 (101–119)	117 (105–132) *
Diastolic blood pressure, mmHg	71 (67–80)	73 (66–80)
Hypertension (by office BP)	32 (41.6%)	17 (48.6%)
Blood urea nitrogen, mg/dL	11 (10–14)	14 (12–18) *
Creatinine, mg/dL	0.53 (0.42–0.62)	0.91 (0.73–1.09) *
eGFR, mL/min/1.73 m^2^	111 (99.7–121.7)	71.6 (56.5–81.6) *
Hemoglobin, g/dL	13.3 (12.8–14)	13.5 (12.4–15.1)
Total cholesterol, mg/dL	170 (144–193)	159 (144–211)
Low-density lipoprotein, mg/dL	93 (74–111)	94 (81–127)
Triglyceride, mg/dL	68 (52–88)	80 (51–103)
Uric acid, mg/dL	4.7 (4–6)	6.8 (5.2–7.6) *
Glucose, mg/dL	90 (85–94)	89 (86–93)
Sodium, mEq/L	140 (139–141)	141 (139–142)
Potassium, mEq/L	4.3 (4.2–4.6)	4.4 (4.2–4.8)
Calcium, mg/dL	9.8 (9.6–10.1)	9.8 (9.7–10.1)
Phosphate, mg/dL	4.6 (4.3–5.1)	4.5 (4–4.9)
Urine total protein-to-creatinine ratio, mg/g	62.7 (35–113.6)	58.9 (25–291.5)

Data are medians (25th, 75th percentile) or *n* (%). eGFR = estimated glomerular filtration rate. * *p* < 0.05 by the Mann–Whitney *U*-test or Chi-square test.

**Table 2:** Urinary levels of acrylamide metabolites in children and adolescents with CKD.

CKD Stage	G1	G2–G4
	*n* = 77	*n* = 35
Acrylamide (ng/mg Cr)	9.25 (5.15–15.2)	5.3 (2.55–11.37) *
AAMA (ng/mg Cr)	31.11 (21.65–51.04)	32.9 (20.97–41.68)
GAMA (ng/mg Cr)	4.7 (3.02–7.21)	3.78 (2.58–5.27)
AAMA-to-GAMA ratio	6.88 (5.63–8.53)	7.92 (6.28–9.8) *

Data are medians (25th, 75th percentile). * *p* < 0.05 by the Mann–Whitney *U*-test. Cr = creatinine. AAMA = N-acetyl-S-(2-carbamoylethyl)-cysteine. GAMA = N-acetyl-S-(2-carbamoyl-2-hydroxyethyl)-cysteine.

**Table 3:** Plasma levels of nitric oxide (NO)-related parameters in children and adolescents with CKD.

CKD Stage	G1	G2–G4
	*n* = 77	*n* = 35
Citrulline	27.25 (18.35–41.43)	32 (24.8–55.2) *
Arginine	90 (55.53–139.25)	92.9 (67–133.8)
ADMA	1.75 (0.9–3.08)	1.8 (1.1–2.7)
SDMA	0.7 (0.4–1.1)	0.9 (0.6–1.4) *
Arginine-to-ADMA ratio	50.65 (36.98–77.35)	48.9 (35.6–84.9)

Data are medians (25th, 75th percentile). * *p* < 0.05 by the Mann–Whitney *U*-test. ADMA = asymmetric dimethylarginine. SDMA = symmetric dimethylarginine.

**Table 4:** Cardiovascular assessments in CKD children and adolescents.

CKD Stage	G1	G2–G4
24 h ABPM	*n* = 48	*n* = 26
Abnormal ABPM profile (with any of the following abnormalities)	23 (47.9%)	16 (61.5%)
Average 24 h BP > 95th percentile	2 (4.2%)	5 (19.2%) *
Average daytime BP > 95th percentile	2 (4.2%)	5 (19.2%) *
Average nighttime BP > 95th percentile	2 (4.2%)	6 (23.1%) *
BP load ≥ 25%	10 (20.8%)	12 (46.2%) *
Nocturnal decrease of BP < 10%	18 (37.5%)	12 (46.2%)
AASI	0.33 (0.21–0.45)	0.43 (0.33–0.58) *
Left ventricular mass (g)	75 (54.6–102)	104 (51.7–142) *
LVMI (g/m^2.7^)	39.8 (32.9–48.7)	42.6 (37.2–47.1)

Data are medians (25th, 75th percentile) or n (%). * *p* < 0.05 by the Mann–Whitney *U*-test. ABPM = 24 h ambulatory blood pressure monitoring. AASI = ambulatory arterial stiffness index. LVMI = left ventricular mass index.

**Table 5:** Correlation between urinary acrylamide metabolite level cardiovascular markers in CKD children and adolescents.

CV Markers	Acrylamide		AAMA		GAMA		AAMA-to-GAMA Ratio	
	*r*	*p*	*r*	*p*	*r*	*p*	*r*	*p*
24 h SBP	−0.368	0.001 *	−0.073	0.535	−0.069	0.557	−0.098	0.406
Daytime SBP	−0.373	0.001 *	−0.084	0.476	−0.082	0.487	−0.091	0.439
Nighttime SBP	−0.356	0.002 *	−0.114	0.334	−0.093	0.431	−0.125	0.288
24 h DBP	−0.32	0.005 *	−0.003	0.978	0.021	0.861	−0.101	0.391
Daytime DBP	−0.31	0.007 *	−0.026	0.824	0.019	0.871	−0.126	0.286
Nighttime DBP	−0.234	0.045 *	0.026	0.823	0.072	0.545	−0.085	0.472
LV mass	−0.566	<0.001 *	−0.34	<0.001 *	−0.559	<0.001 *	0.261	0.005 *
LVMI	0.035	0.718	0.002	0.98	0.014	0.887	−0.096	0.316
AASI	−0.02	0.866	0.160	0.173	−0.004	0.974	0.252	0.03 *

AAMA = N-acetyl-S-(2-carbamoylethyl)-cysteine. GAMA = N-acetyl-S-(2-carbamoyl-2-hydroxyethyl)-cysteine. SBP = systolic blood pressure. DBP = diastolic blood pressure. LVMI = left ventricular mass index. AASI = Ambulatory arterial stiffness index. * *p* < 0.05 by Spearman’s correlation coefficient.

**Table 6:** Urinary acrylamide metabolite levels vs. ABPM profile in CKD children and adolescents.

ABPM Profile	*n*	Acrylamide	AAMA	GAMA	AAMA-to-GAMA Ratio
		ng/mg Cr	ng/mg Cr	ng/mg Cr	ng/ng
24 h BP					
Abnormal	7	4.87 (3.76–10.81)	39.49 (20.97–50.86)	4.6 (3.28–5.27)	7.55 (6.14–8.59)
Normal	67	6.7 (3.28–11.2)	28.37 (19.3–37.3)	3.54 (2.64–5.04)	7.48 (6.19–9.62)
Daytime BP					
Abnormal	7	4.87 (3.76–10.81)	39.49 (20.97–50.86)	4.6 (3.28–5.27)	7.55 (6.14–8.59)
Normal	67	6.7 (3.28–11.2)	28.37 (19.3–37.3)	3.54 (2.64–5.04)	7.48 (6.19–9.62)
Nighttime BP					
Abnormal	8	5.01 (4.02–9.91)	29.34 (21.12–39.67)	3.61 (2.97–5.1)	7.54 (6.48–8.42)
Normal	66	6.51 (3.26–11.24)	28.46 (19.21–40.71)	3.61 (2.63–5.08)	7.48 (6.19–10.05)
BP load					
Abnormal	22	4.84 (2.54–6.83) *	28.87 (18.76–42.33)	3.61 (2.55–4.76)	7.52 (6.13–8.98)
Normal	52	7.69 (4.52–12.88)	28.35 (19.84–40.31)	3.61 (2.69–5.1)	7.42 (6.2–9.6)
Night dipping					
Abnormal	30	5.09 (2.54–8.17) *	28.32 (18.45–39.95)	3.72 (2.4–4.82)	8 (6.17–9.67)
Normal	44	7.64 (4.59–12.71)	29.64 (20.27–40.31)	3.52 (2.7–5.16)	7.38 (6.23–9.31)
ABPM profile					
Abnormal	39	5.05 (2.64–9.52) *	28.32 (18.45–39.95)	3.72 (2.4–4.82)	8 (6.17–9.67)
Normal	35	8.84 (4.59–12.85)	30.9 (19.6–40.5)	3.54 (2.69–5.11)	7.27 (6.27–10.77)

Data are medians (25th, 75th percentile) or n (%). AAMA = N-acetyl-S-(2-carbamoylethyl)-cysteine. GAMA = N-acetyl-S-(2-carbamoyl-2-hydroxyethyl)-cysteine. * *p* < 0.05 by the Mann–Whitney *U*-test.

**Table 7:** Adjusted regression model estimates of the association between urinary acrylamide metabolites and NO pathway and cardiovascular (CV) risk markers in CKD children and adolescents.

Dependent Variable	Explanatory Variable	Adjusted ^a^		Model	
		Beta	*p* Value	*r*	*p* Value
Citrulline	AAMA	−0.831	0.006	0.45	0.001
	GAMA	1.126	<0.001		
	AAMA-to-GAMA ratio	0.309	0.022		
SDMA	GAMA	0.608	0.047	0.39	0.015
LVMI	AAMA	−0.717	0.015	0.472	<0.001
	GAMA	0.715	0.014		
	AAMA-to-GAMA ratio	0.27	0.041		

^a^ Adjusted for age, sex, and eGFR. SDMA = symmetric dimethylarginine; LVMI = Left ventricular mass index; AAMA = N-acetyl-S-(2-carbamoylethyl)-cysteine; GAMA = N-acetyl-S-(2-carbamoyl-2-hydroxyethyl)-cysteine.

## Abbreviations

AAMA	N-acetyl-S-(2-carbamoylethyl)-cysteine
AASI	Ambulatory arterial stiffness index
ABPM	24 h ambulatory blood pressure monitoring
ADMA	Asymmetric dimethylarginine
CKD	Chronic kidney disease
CVD	Cardiovascular disease
eGFR	Estimated glomerular filtration rate
GAMA	N-acetyl-S-(2-carbamoyl-2-hydroxyethyl)-cysteine
GSH	Glutathione
LVMI	Left ventricular mass index
SDMA	Symmetric dimethylarginine
